# Effect on Neuropsychiatric Inventory–Questionnaire scores in the National Alzheimer's Coordinating Center Uniform Data Set

**DOI:** 10.1002/alz70857_098595

**Published:** 2025-12-24

**Authors:** Juan‐Camilo Vargas‐González, Martin Ingelsson, David F. Tang‐Wai, Carmela Tartaglia

**Affiliations:** ^1^ University Health Network, Toronto, ON, Canada; ^2^ Memory Clinic, Toronto Western Hospital, University Health Network, Toronto, ON, Canada; ^3^ Temerty Faculty of Medicine, University of Toronto, Toronto, ON, Canada; ^4^ University of Toronto, Toronto, ON, Canada

## Abstract

**Background:**

Neuropsychiatric symptoms (NPS) are frequently encountered in neurodegenerative diseases including Alzheimer's disease (AD). The Neuropsychiatric Inventory–Questionnaire (NPI‐Q) is the most frequently used assessment for NPS in patients with AD. It is an informant‐ based assessments wherein informants are asked to rate NPS in the patient. We have found that informant characteristics influence the Clinical Dementia Rating (CDR) in the National Alzheimer's Coordinating Center Uniform Data Set (NACC‐UDS). We aimed to evaluate informant characteristics on the NPI‐Q in patients with mild cognitive impairment or dementia due to AD participating in the NACC‐UDS.

**Method:**

We included all participants from the NACC‐UDS that had AD as the principal diagnosis, and information about the number of Clinician Judgments of Behavioural Symptoms (CJbS), informant characteristics, CDR global score (CDR‐GS) and the outcome NPI‐Q severity score. We performed a conditional growth model using multilevel linear regression analysis.

**Result:**

We included 21276 participants, totalling 56301 visits with a median of 3 (1‐5) visits. Patients' age at the initial visit was 74.0±9.4 years and 54.1% were females. Informants were 66.2±13.1 years, 68.7% were females, and the relationship was 60.4% spouse or partner, 27.7% children and 11.9% other.

The NPI‐Q scores were affected by informant characteristics. The associations were 0.39 (CI 95%:0.31 to 0.46) higher with female informants. The NPI‐Q was 0.34 (CI 95%: 0.23 to 0.45) higher when informants were children of the patients compared with spouses or partners. The frequency of visits was associated with an NPI‐Q score 0.25 lower (CI 95%:‐0.39 to ‐0.11) when visiting daily, 0.53 lower (CI 95%:‐0.64 to ‐0.42) when visiting at least once per week, and 0.60 lower (CI 95%:‐0.74 to ‐0.46) when visiting less than once a week compared with living with the patient. As expected, NPI‐Q scores increased with higher CDR‐GS and CJbS.

**Conclusion:**

We found that the NPI‐Q severity scores are modified by informant characteristics in the NACC‐UDS patients with AD diagnosis. These results are clinically relevant because NPI‐Q is often used to inform treatment of NPS in patients with AD and informant characteristics are not considered when undertaking treatments.

